# Dosimetric impact of mechanical movements of the Linac gantry during treatments with small fields

**DOI:** 10.3389/fonc.2022.973431

**Published:** 2022-11-03

**Authors:** Broderick Ivan McCallum-Hee, Thomas Milan, Rohen White, Pejman Rowshanfarzad

**Affiliations:** ^1^ School of Physics, Mathematics and Computing, The University of Western Australia, Crawley, WA, Australia; ^2^ Department of Radiation Oncology, Sir Charles Gairdner Hospital, Nedlands, WA, Australia

**Keywords:** quality assurance, stereotactic, small field, VMAT, mechanical isocentre, gantry movement, linear accelerator

## Abstract

**Objective:**

Current accepted linac Quality Assurance (QA) guidelines used for Volumetric Modulated Arc Therapy (VMAT) suggest a mechanical isocentre tolerance level of 1 mm. However, this tolerance level has not been well-established for the specific case of small field stereotactic VMAT. This study aims to evaluate the clinical impact of mechanical uncertainty on this treatment modality by modelling systematic gantry sag derived isocentre variance in the Treatment Planning System (TPS).

**Approach:**

A previously reported dataset of gantry sag values in the literature served as a starting point for this study. Using an in-house developed VMAT arc splitting algorithm, isocentre shifts were applied at a Control Point (CP) level to DICOM-RT treatment plans. Dose distributions for varying isocentre shift magnitudes were calculated for a set of 29 stereotactic VMAT plans using the Eclipse Acuros XB dose algorithm. These plans had a range of Planning Target Volume (PTV) sizes. A quantitative comparison of each plan was conducted by evaluating five Dose Volume Histogram (DVH)-derived plan quality metrics.

**Results:**

All metrics exhibited a deterioration in plan quality with increasing magnitudes of isocentre shift. At small PTV sizes, these effects were amplified, exhibiting significant changes at 1 mm of average shift when typical targets and tolerances were considered. For plans with PTVs between 0 and 5 cm^3^, a 1 mm shift reduced PTV coverage by 6.6 ± 2.2% and caused a 12.1 ± 3.8% deterioration in the conformity index. Based on the results of this study, the prevalent tolerance of 1 mm may not be suitable for treatments of small PTVs with small fields.

**Significance:**

In contrast to commonly accepted values, an absolute mechanical isocentre of 0.5 mm with action level at 0.75 mm is recommended for stereotactic VMAT of PTV sizes below 10 cm^3^.

## Introduction

Volumetric Modulated Arc Therapy (VMAT) is a radiotherapy technique which is used to produce highly conformal dose distributions in relatively short delivery times compared to older conformal methods, such as IMRT (1). This technique is increasingly delivered using small fields as stereotactic techniques, such as Stereotactic Ablative Radiotherapy (SABR) or Stereotactic Body Radiotherapy (SBRT), become more prevalent and Multileaf Collimator (MLC) access improves (2). Although CyberKnife or GammaKnife can also provide stereotactic radiotherapy, the gantry-based medical linear accelerator (linac) is still the most frequently used machine in external beam radiotherapy (3). Routine linac Quality Assurance (QA) is critical to achieving optimal patient outcomes, particularly for high precision techniques, such as stereotactic VMAT.

In linac QA, one of the many critical parameters which must be monitored is the stability of the mechanical isocentre (4). It is well established that imperfections in linac gantry rotation caused by the strong pull of gravity lead to slight deviations to the isocentre during treatment delivery. Several studies have investigated this effect, and its angular dependence has been well quantified (5–8). Although QA programs are designed at an institution level, they are informed by vendor specifications as well as recommendations from the literature and professional organisations such as the American Association of Physicists in Medicine (AAPM).

Denton et al. conducted a statistically driven examination of isocentre congruency tests collected over a year to propose practical mechanical isocentre tolerance levels for SBRT (9). An absolute schedule action level of 1.25 mm and an immediate action level of 1.5 mm were proposed. Tsai et al. outlined a clinically implemented Stereotactic Radiosurgery (SRS) QA program in which the absolute mechanical isocentre tolerance is 0.5 mm (10). The ubiquitous AAPM TG-142 Report and following AAPM TG-198 Report recommend a mechanical isocentre tolerance of 1 mm from baseline for VMAT with no further stringent stereotactic treatment recommendations (4, 11).

Milan et al. investigated the impact of gantry and MLC carriage sag on VMAT clinical performance for Elekta and Varian linacs (12). They manipulated Digital Imaging and Communications in Medicine (DICOM) files by splitting VMAT arcs into sub-beams before dose calculation *via* Monte Carlo simulation. In all plans processed, increasing isocentre shifts resulted in a deterioration of plan quality. Despite this, minimal reductions in Planning Target Volume (PTV) coverage were observed for less than 1 mm mechanical isocentre shift, supporting the TG-142 acceptance criterion. Similarly, investigation of the dose difference global function and gamma index was in support of TG-142.

Wack et al. investigated the impact of isocentre shifts due to linac gantry and table rotation during cranial conformal static stereotactic treatment (13). They considered measured isocentre variations for two linacs and conducted a planning study using Pinnacle^3^. In the planning study a series of hypothetical spherical PTVs with ten noncoplanar conformal static beams were generated, and uniform isocentre shifts applied. Results suggested a 1 mm threshold for isocentre shift may be too large for stereotactic irradiation of very small target volumes less than 2 cm^3^. A recommended level of 0.5 mm was suggested for which minimal plan deterioration was observed.

This study aims to evaluate, for the first time, the effect that varying magnitudes of mechanical isocentre uncertainty have on small field VMAT plan performance. Measured sag results from Rowshanfarzad et al. (6–8) provided a starting point for the work, which took a computational approach consisting of DICOM modification, dose simulation and quantitative dose comparison using clinical plan performance metrics. This work provides essential data on the suitability of current QA practices and enables more informed decisions.

## Materials and methods

### Gantry sag data

Quantified gantry sag data for various Varian, Elekta and Siemens linacs from Rowshanfarzad et al. (6–8) considers different linac models and ages. All average sag patterns were similar despite variance in each linac. The gantry sag for a specific machine depended on many factors, including machine type, age, and specific setup. While these patterns provide a helpful reference, a ‘real-world’ linac may present differently.

In this study, the Elekta gantry sag pattern from Rowshanfarzad et al. (6) was taken as representative of measured data to be applied in a machine agnostic methodology. While this pattern’s differences from other machines were noted, such as a smoother cross-plane gantry sag pattern over the zero angle than that for Varian machines, each linac is unique. Further, the effect of sag magnitude differences was not relevant as varying magnitudes of the pattern were considered. The Elekta data was provided as a pre-interpolated continuous Fourier series across the full range of gantry motion (6).

### Isocentre modification

**Figure 1:**
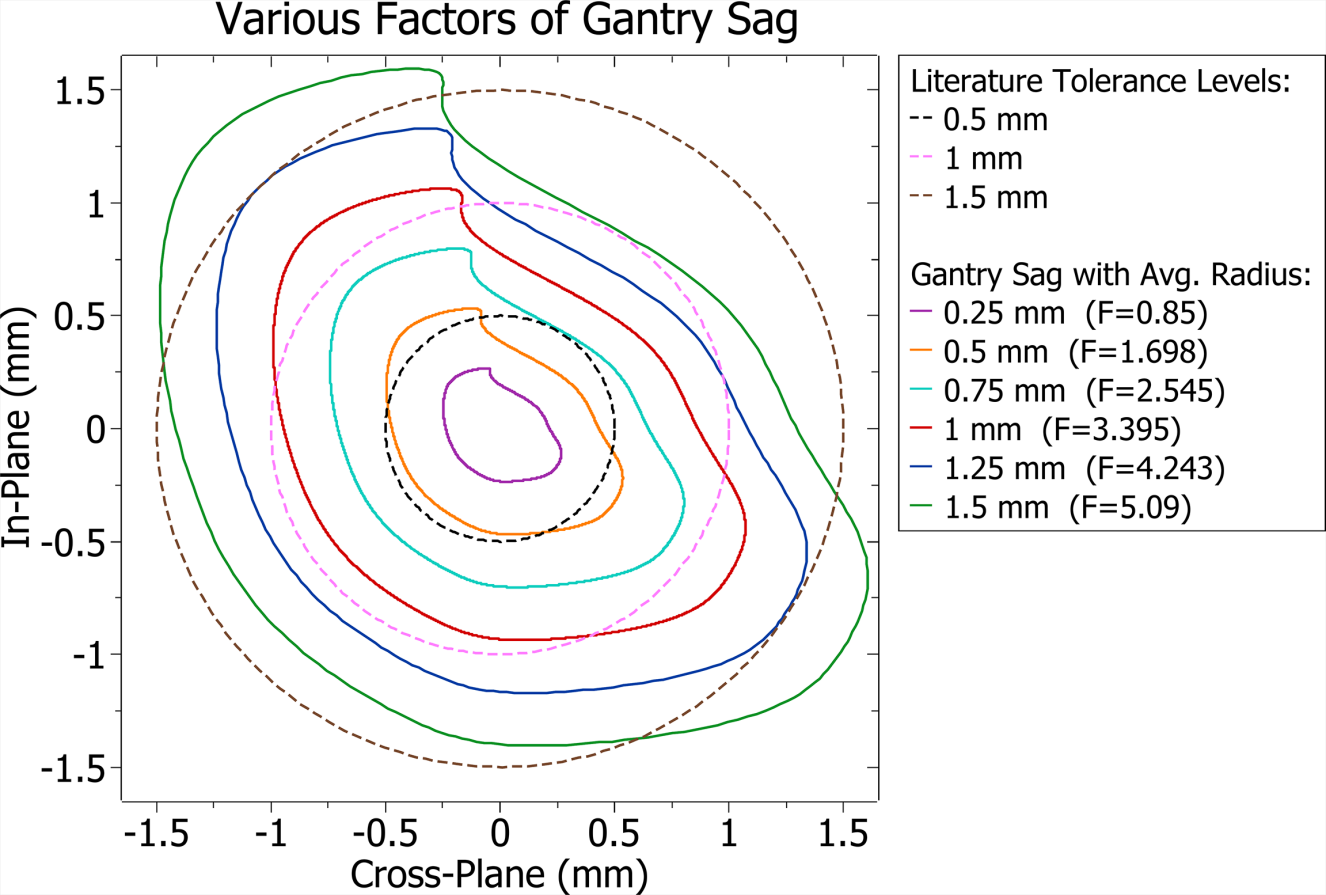
Cross-plane and in-plane gantry sag patterns with varying scaling factors (F).

Gantry sag was modelled by modifications to the isocentre, and other contributions such as couch and collimator sag were not considered. The average distance the isocentre was from its intended position at any given point was found for various scale factors (F) of the Elekta data. Factors were found such that the average distances of the isocentre shift were equal to different QA tolerance levels found in literature and midpoints between them. The reference tolerance levels and gantry sag patterns at varying scaling factors and their average radius are shown in **Figure 1**.

### DICOM modification

Stereotactic VMAT plan DICOM files were imported into MATLAB (version 9.9.0 R2020b, The MathWorks Inc.) for manipulation. This process enabled complex plan modifications which were re-written back into DICOM format.

**Figure 2:**
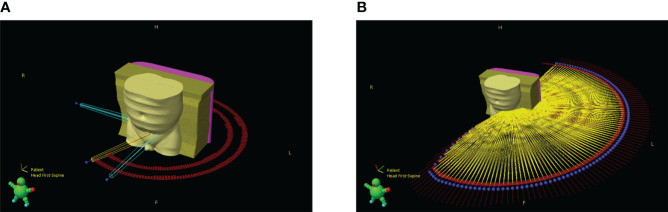
3D render of patient and beam geometry **(A)** before and **(B)** after the splitting process. Blue represents setup beams, yellow treatment beams, red marks are used to outline control point positions and the blue spheres represent the starting point of a beam.

Although the gantry sag data enables derivation of gantry sag attributed isocentre shift at any angle, during VMAT treatment planning, arc instructions are given at a control point (CP) level. The effect of these shifts can therefore only be assessed to this accuracy. In VMAT dose is delivered between CPs, and the isocentre in the DICOM file must be stable between them, or the treatment planning system (TPS) will reject it. This issue was circumvented by splitting each VMAT arc at every CP. The splitting process creates a series of new shorter VMAT arcs for which their combined effect is equivalent to the original arc, as shown in **Figure 2**. Avoiding the issues encountered by Milan et al. when a similar process was employed, the Varian Eclipse TPS (used in this study) has no beam limit.

The effect of beam splitting is briefly discussed in the results. However, as this work investigated the relative dosimetric effect of applied shifts at varying magnitudes, absolute congruency with prior to split plans was not required if dose distributions were closely comparable.

**Figure 3:**
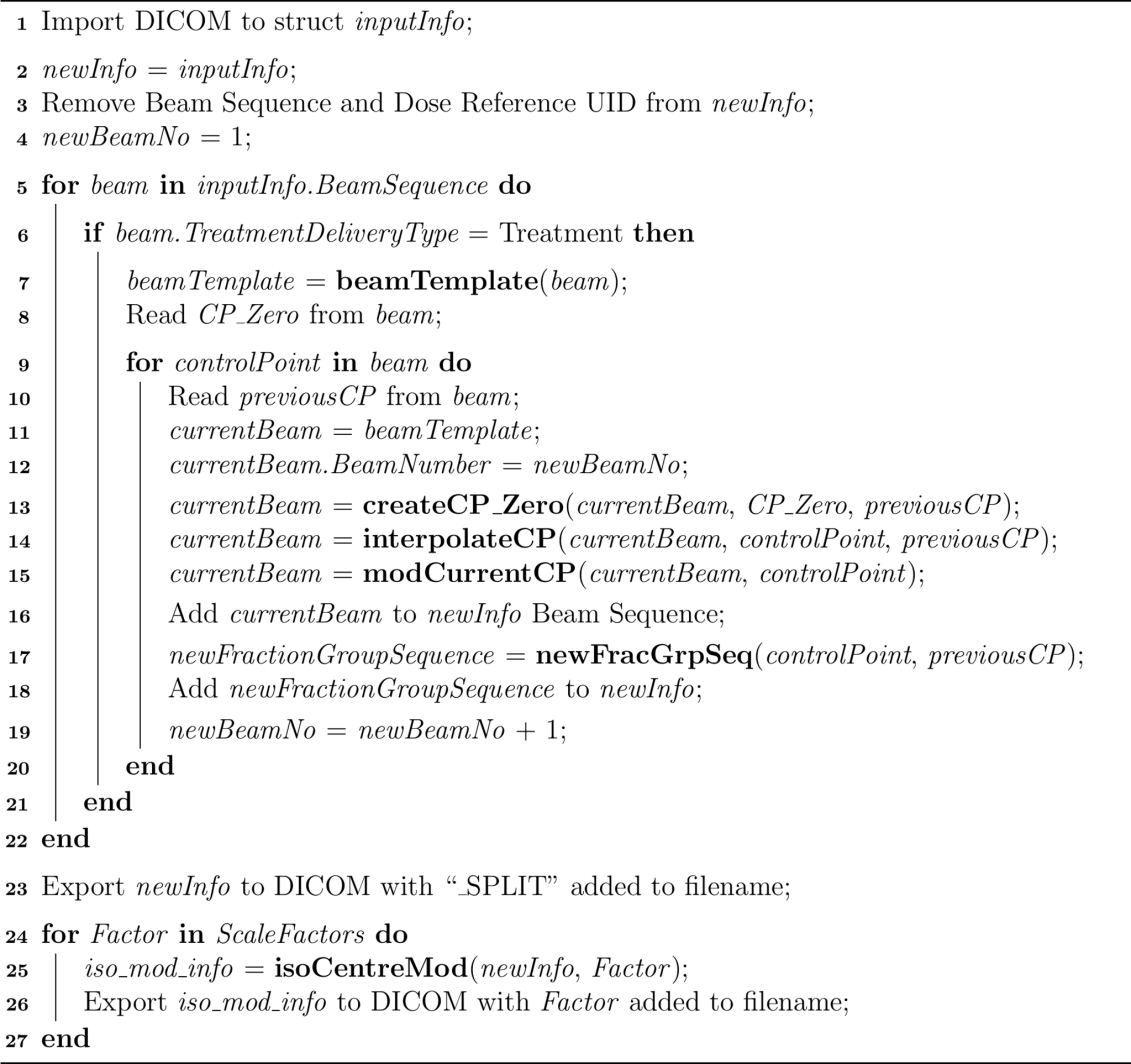
Overview of MATLAB Script Structure. Lines 1 – 4 remove plan identifiers and setup required objects. Lines 5 – 22 split the VMAT arcs at each CP, linearly interpolate midpoints and modify the fraction group sequence such that the overall monitor units remain the same. Line 23 exports the split plan to DICOM. Lines 24 – 27 apply varying magnitudes of isocentre shift and export the produced plans to DICOM.

An overview of the MATLAB script created to modify plan DICOM files is included in **Figure 3**. The script removes plan identifying attributes, splits the VMAT arcs and applies the varying factors of gantry sag shown in **Figure 1** by changing the isocentre accordingly at each beam angle. Although isocentre shifts may affect the accuracy of setup beams and subsequently indirectly change treatment quality, it was considered sufficient to only consider dosimetric impacts *via* treatment beams. During Eclipse TPS MLC movement processing, a minimum of three CPs is required. To support this, each of the split VMAT arcs contained modified versions of the respective original two CPs and a linearly interpolated third midpoint.

### Dose calculation

Dose calculations were conducted in the Varian Eclipse TPS using Acuros Version 16.1.0, which is a highly accurate linear Boltzmann transport equation-based algorithm that can produce dose distributions comparable to Monte Carlo methods for small fields (14). A dose calculation grid of 1.25 mm was used, which is well within the recommended maximum of 2 mm for stereotactic plans (15, 16).

### Dose analysis

To investigate the impact of scaling factors of isocentre shift five metrics were considered: PTV coverage, PTV dose near-minimum, prescription dose spillage, gradient index, and conformity index. Scale factors of 0.85, 1.698, 2.545, 3.395, 4.243 and 5.09 were applied, allowing trends to be investigated. However, to discuss the response of these dose metrics in terms of shift magnitude, it was decided to use the average shift (in mm) rather than a dimensionless scale factor.

The PTV dose near-minimum and conformity index are recommended by the International Commission on Radiation Units (ICRU) for reporting stereotactic treatments with small fields (17). The dose near-minimum was reported as a percentage of the prescribed dose. The inverse of Paddick’s conformity index, referred to here as the conformity index, is defined as:


$$\text{Conformity Index=}\frac{TV\times PIV}{TV_{PIV}^{2}}$$

Where TV is the target volume, PIV is the prescription isodose volume and TV_PIV_ is the TV within the PIV.

A modified version of prescription dose spillage and gradient index is recommended for stereotactic treatment planning by the SABR UK Consortium (15). These metrics are defined:


$$\text{Prescription Dose Spillage = }\frac{PIV}{TV_{PIV}}$$

$$\text{Gradient Index = }\frac{PIV_{50}}{TV_{PIV}}$$

where PIV_50_ is the volume receiving at least half the prescription dose.

### Plans

A set of 28 stereotactic VMAT plans were examined in this study. The data set included pelvic bone, brain and femur treatment sites. The majority were pelvic bone treatments. A summary of plans and their key characteristics is included in **Table 1**.

**Table 1:** Summary of all plans used in this study and their key characteristics grouped by PTV size.

PTV Category	GTV (cm^3^)	CTV (cm^3^)	PTV (cm^3^)	Treatment Site	Dose (Gy)/Fractions (# × Gy)	Beams
0 - 5	–	–	1.41	Brain	12 (1 × 12)	2 × 360°
	0.41	–	2.4	Brain	12 (1 × 12)	2 × 360°
	1.62	–	2.78	Brain	12 (1 × 12)	3 × 360°
	0.21	–	3.04	Pelvis - Iliac (Right)	21 (3 × 7)	2 × 200°
	0.2	–	3.16	Pelvis - Sacrum	30 (5 × 6)	2 × 180°
	0.58	–	3.22	Femur (Left)	27 (3 × 9)	2 × 230°
	1.51	–	4.41	Pelvis - Iliac (Left)	24 (3 × 8)	2 × 200°
5 - 10	0.54	–	5.64	Pelvis - Ilium (Right)	8 (1 × 8)	2 × 200°
	1.38	3.25	6.72	Pelvis - Pubis (Right)	30 (5 × 6)	2 × 360°
	1.21	4.34	8.38	Pelvis - Ilium (Left)	35 (5 × 7)	2 × 220°
10 -15	0.61	5.03	11.81	Pelvis - Ischium (Left)	27 (3 × 9)	2 × 190°
	2.37	6.37	11.93	Pelvis - Ilium (Right)	35 (5 × 7)	2 × 190°
	3.63	–	12.89	Pelvis - Iliac (Left)	30 (5 × 6)	2 × 200°
	1.47	4.95	14.11	Pelvis - Ischium (Left)	35 (5 × 7)	2 × 360°
	1.68	4.79	14.78	Scapula (Right)	35 (5 × 7)	2 × 210°
15 - 35	10.73	–	20.85	Pelvis - Iliac (Left)	30 (5 × 6)	2 × 220°
	5.63	9.98	20.97	Pelvis - Iliac (Right)	35 (5 × 7)	2 × 210°
	13.07	–	24.03	Pelvis - Iliac (Right)	25 (5 × 5)	2 × 180°
	1.7	17.67	33.56	Pelvis - Ischium (Left)	20 (5 × 4)	2 × 200°
	1.89	18.38	34.84	Pelvis - Pubic Ramus (Left)	30 (5 × 6)	2 × 200°
35 - 55	14.2	–	40.62	Pelvis - Ischium (Left)	30 (5 × 6)	2 × 180°
	21.17	–	40.75	Pelvis - Sacrum	15 (1 × 15)	3 × 360°
	3.85	25.75	45.8	Pelvis - Pubis (Left)	27 (3 × 9)	3 × 180°
	28.12	–	47.29	Pelvis - Sacrum (Nodes)	30 (5 × 6)	2 × 360°
	9.3	12.14	47.91	Scapula (Left)	27 (3 × 9)	2 × 160°
55 +	19.39	34.84	58.95	Pelvis - Sacrum	30 (5 × 6)	2 × 360°
	12.62	51.51	85.24	Pelvis - Acetabulum (Left)	27 (3 × 9)	3 × 180°
	5.14	56.79	89.09	Neck of Femur (Left)	27 (3 × 9)	3 × 180°

## Results

Eight dose distributions were calculated for each of the 28 plans summarised in **Table 1**. These distributions were for the original unmodified plan, VMAT arc split version and six magnitudes of isocentre shift. In total, 224 dose distributions were calculated and their metrics extracted. As the starting value of each metric varied between plans, all values were normalised.

The DVHs at different radii of isocentre shift from three treatment sites are shown in **Supplementary Material Figures S1 – S3**. **Table S1** in the **Supplementary Material** shows that GTV coverage was minimally reduced for the shifts evaluated.

The Shapiro-Wilk normality test confirmed that the data followed a normal distribution. As such, t-tests were used to check for statistical significance where required. P-values are reported as “(P = X)”, where X is the P-value.

### Validation of beam splitting

**Table 2** shows the overall average differences between original and split plans to three significant figures. The three brain plans exhibited the most variance post-splitting process, with one (PTV = 1.41 cm^3^) exhibiting the most significant of all plans with a change in PTV coverage of 9.86%. If the brain plans were excluded, no metric’s average percentage difference exceeded 0.08%. These changes indicated the relative congruency between the original and VMAT arc split plans.

**Table 2:** Overall average and percentage differences between original and split treatment plans.

Original vs. Split	PTV Coverage	PTV Near-Minimum/Prescription Dose	Prescription Dose spillage	Gradient Index	Conformity Index
Difference	0.01129	0.00610	0.00580	0.04731	0.01039
% Difference	1.27%	0.66%	0.59%	1.13%	1.00%

### PTV coverage

**Figure 4:**
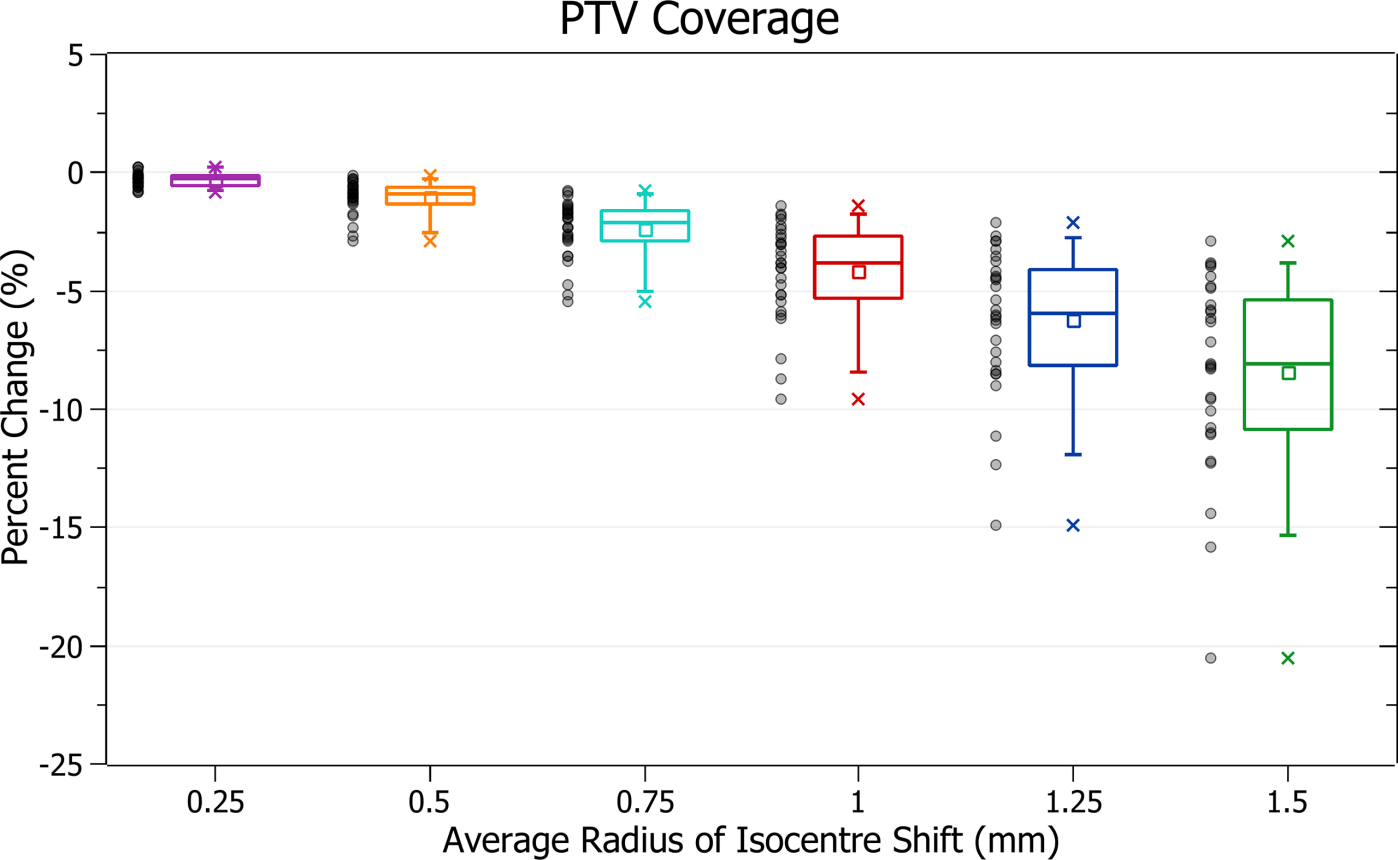
Percentage change of PTV coverage for different radii of isocentre shift.

**Figure 5:**
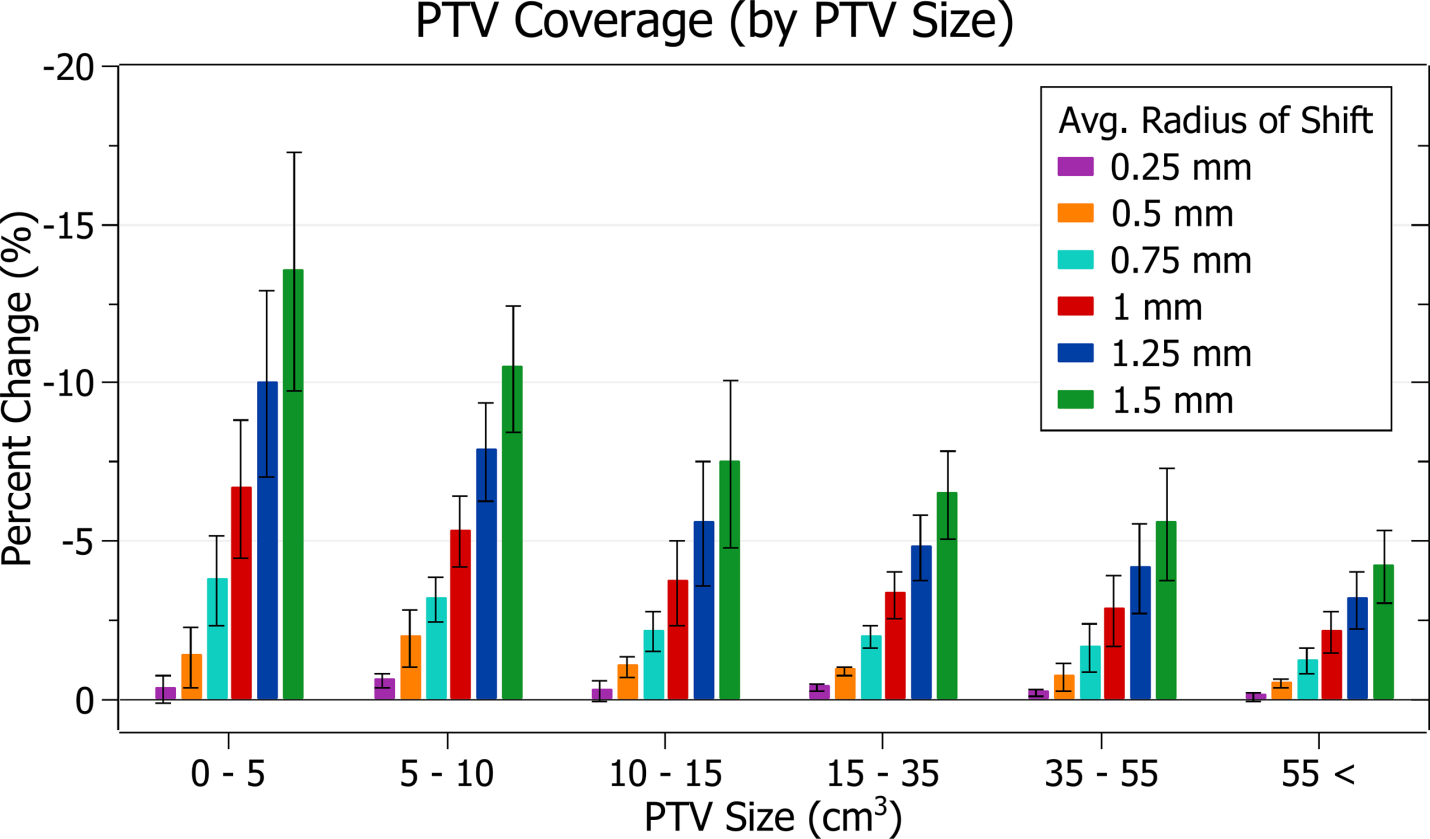
Average percentage change of PTV coverage for different radii of isocentre shift, categorised by PTV size.

The results of PTV coverage against radius of isocentre shift are plotted in **Figure 4**. Categorised averages of these shifts are plotted in **Figure 5** for PTV size.

**Figure 4** shows a decrease in coverage with increasing radii. At 0.25 mm the decrease is 0.3 ± 0.3% (P = 8E-6), 0.5 mm: 1.1 ± 0.7% (P = 1E-8), 1 mm: 4.2 ± 2.1% (P = 4E-11) and 1.5 mm: 8.4 ± 4.1% (P = 3E-11). The three data points exhibiting the most significant decrease for each data set, including the outliers, are from the small PTV brain treatment plans.

Smaller PTVs were closely correlated with increased sensitivity for a given radius of isocentre shift, as depicted in **Figure 5**. For plans with PTVs between 0 and 5 cm^3^ the largest decreases were observed with 13.5 ± 3.8% for 1.5 mm isocentre shift and 6.6 ± 2.2% for 1 mm. At an average shift of 1 mm, for PTVs between 5 - 10, 10 - 15 and 15 - 35 cm^3^ the average percent decreases were 5.3 ± 1.1%, 3.7 ± 1.4% and 3.3 ± 0.7%, respectively.

### PTV near-minimum

**Figure 6:**
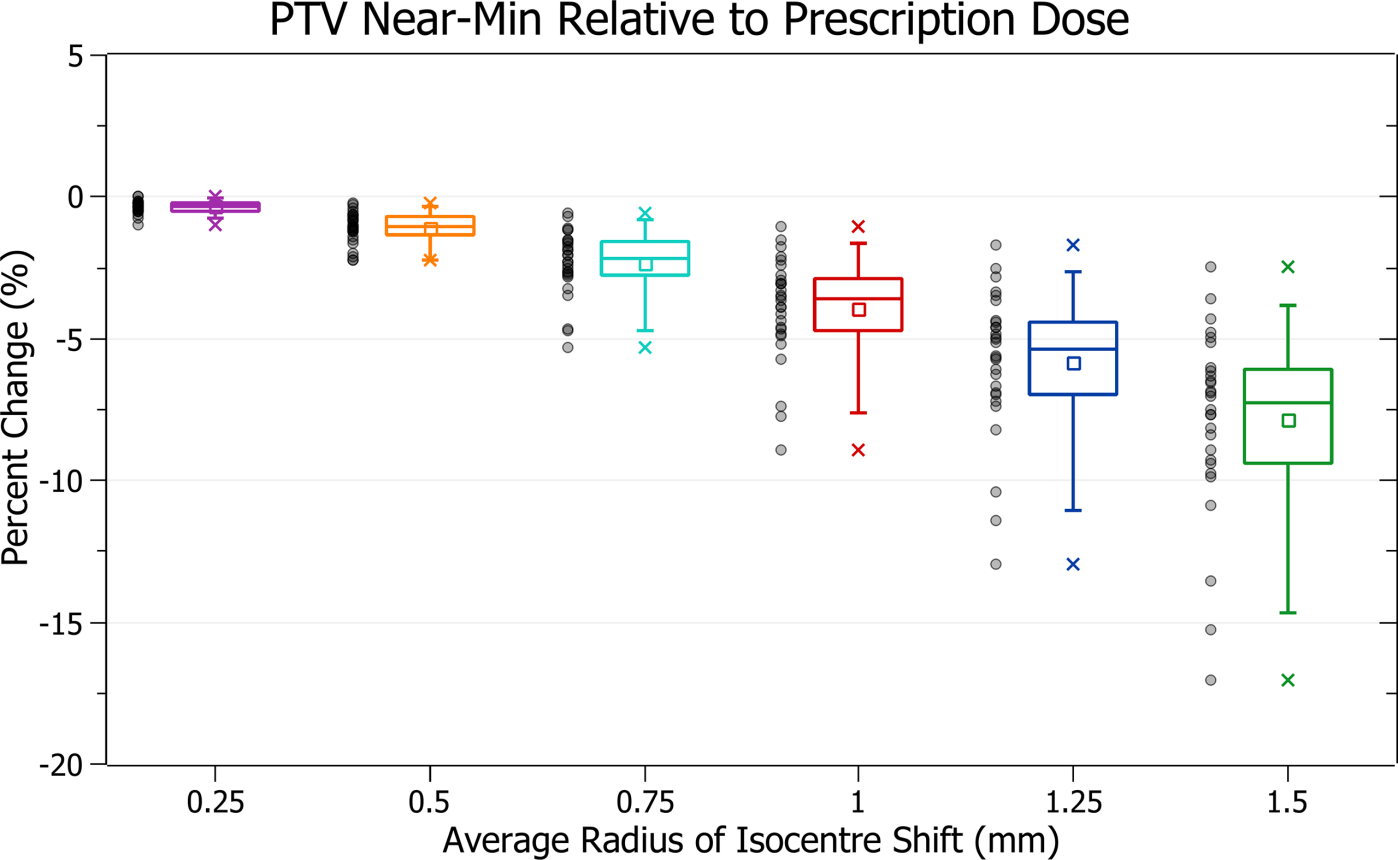
Percentage change of PTV near-min relative to prescription dose for different radii of isocentre shift.

**Figure 7:**
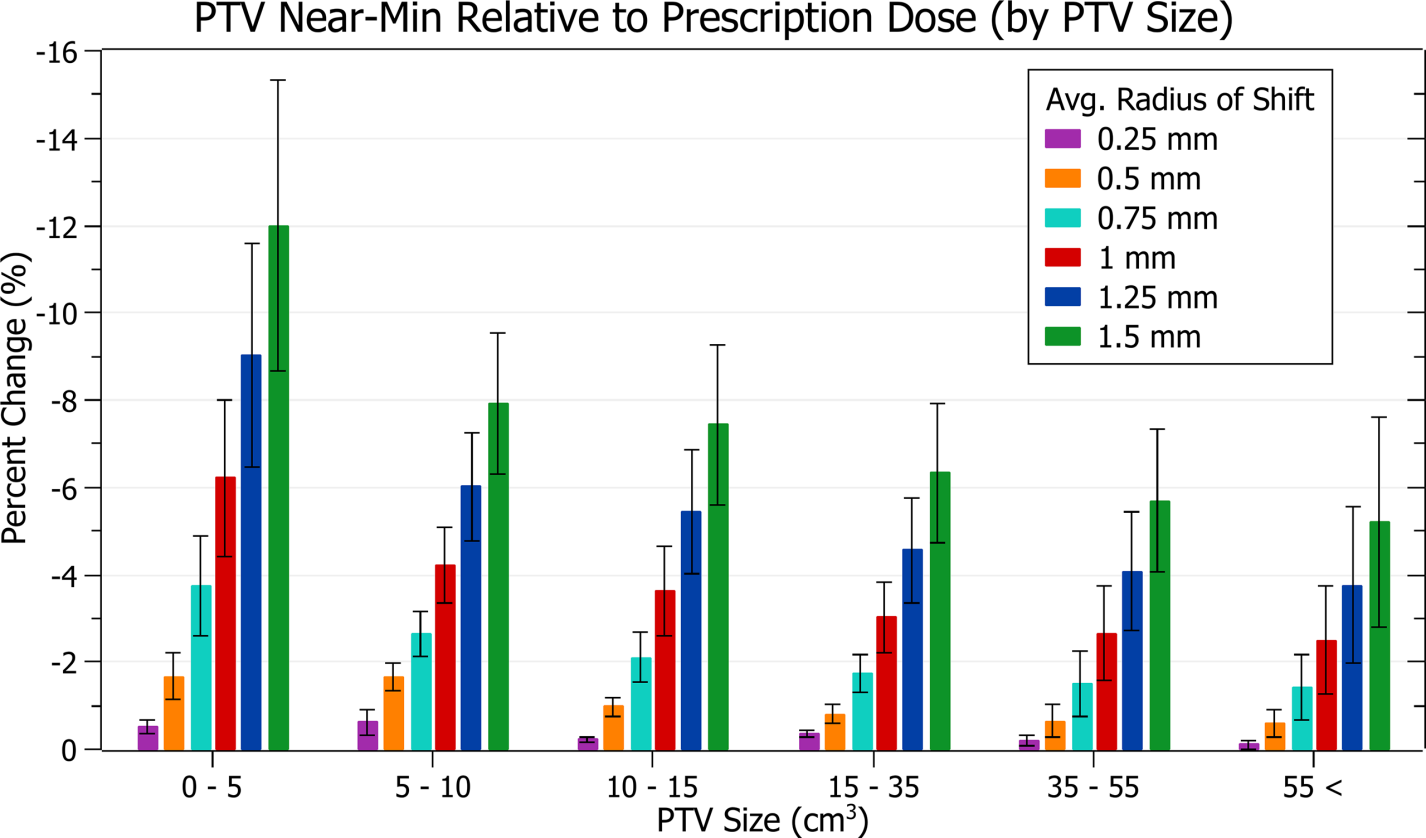
Average percentage change of PTV near-min relative to prescription dose for different radii of isocentre shift, categorised by PTV size.

The results of PTV near-min relative to prescription dose against radius of isocentre-shift are plotted in **Figure 6**. Categorised averages of these shifts are plotted in **Figure 7** for PTV size.

**Figure 6** shows a decrease in PTV near-min dose with increasing isocentre shift, similar to PTV coverage reduction. At 0.5 mm this decrease is 1.1 ± 0.6% (P = 9E-11), 1 mm: 3.9 ± 1.8% (P = 9E-12) and 1.5 mm: 7.9 ± 3.3% (P = 8E-13). The three data points exhibiting the most significant decrease are also similarly from the brain treatments.

In **Figure 7**, smaller PTVs also correlated with increased sensitivity, although the trend was slightly less smooth. At an average shift of 1 mm, the average percentage decreases for PTVs between 0 - 5, 5 - 10 and 10 - 15 cm^3^ were 6.2 ± 1.8%, 4.2 ± 0.9% and 3.6 ± 1.0%. The changes between PTVs 0 - 5 and 5 - 10 were sharper than for PTV coverage resulting in a less smooth overall trend.

### Prescription dose spillage

**Figure 8:**
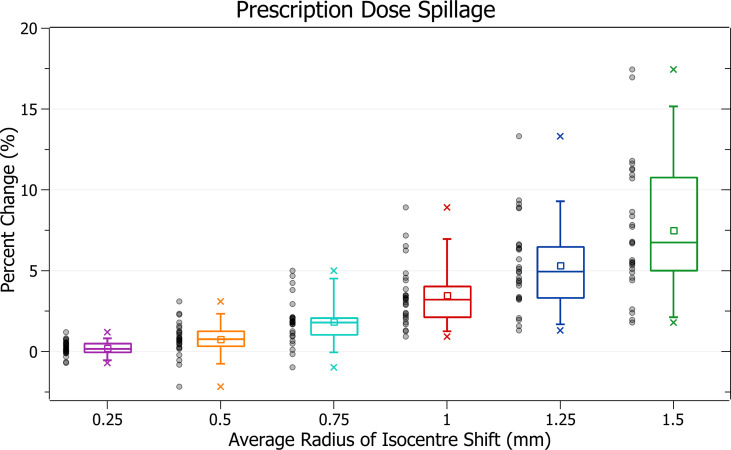
Percentage change of prescription dose spillage for different radii of isocentre shift.

**Figure 9:**
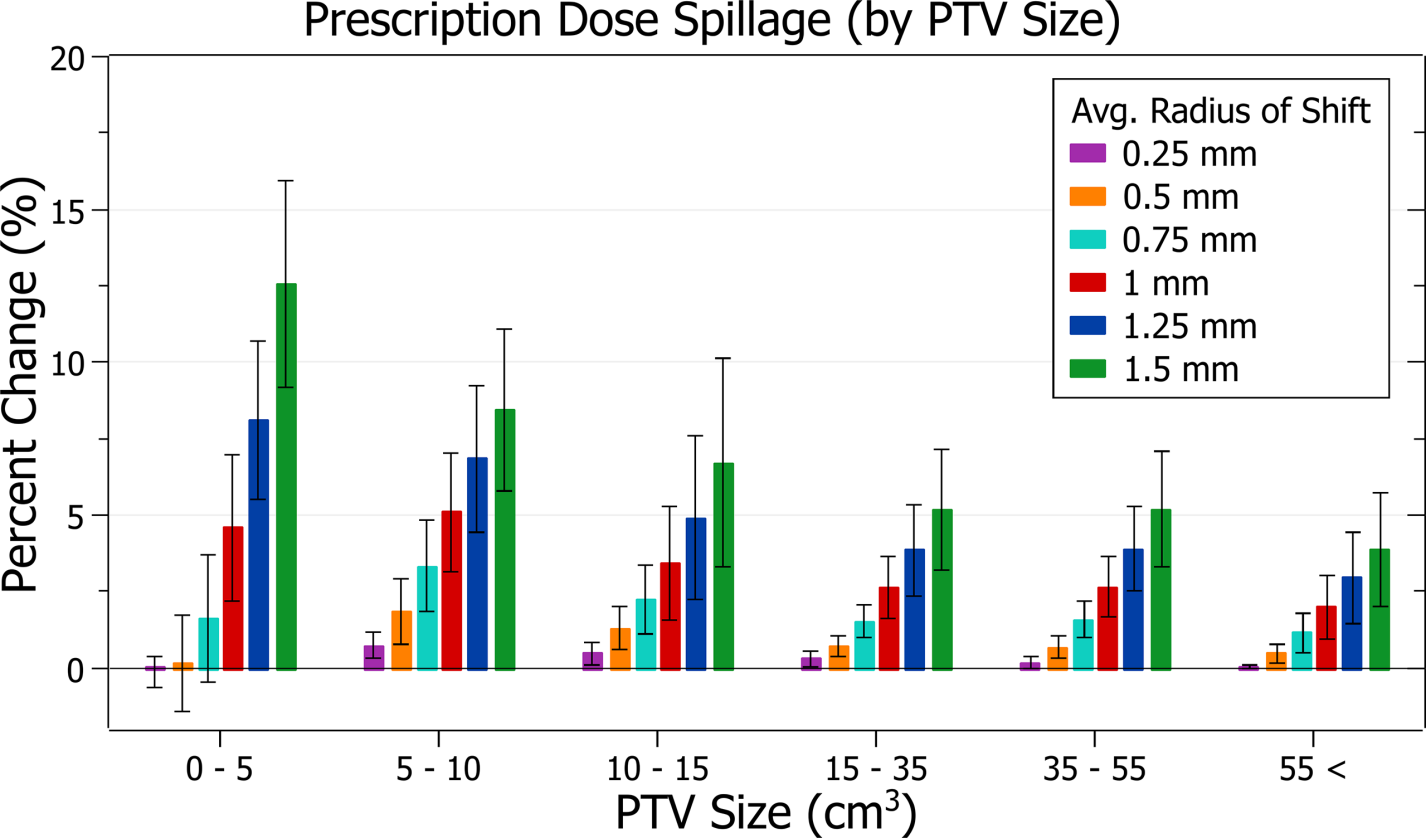
Average percentage change of prescription dose spillage for different radii of isocentre shift, categorised by PTV size.

The results of prescription dose spillage against radius of isocentre shift are plotted in **Figure 8**. Categorised averages of these shifts are plotted in **Figure 9** for PTV size.

**Figure 8** exhibits the increase in spillage with increased shifts. At 0.5 mm the increase is 0.76 ± 1.02% (P = 5E-4), 1 mm: 3.46 ± 1.88% (P = 3E-10) and 1.5 mm: 7.49 ± 4.05% (P = 2E-10). The plans exhibiting the most significant spillage were the brain treatments.

In **Figure 9**, smaller PTV size generally correlated with increased isocentre shift sensitivity; however, there were some variations. At an average shift of 1 mm, the average percent increases for PTVs between 0 - 5, 5 - 10, 10 - 15 and 15 - 35 cm^3^ were 4.6 ± 2.4%, 5.1 ± 2.0%, 3.4 ± 1.9% and 2.6 ± 1.0%, respectively. Notably, the average increase for the 5 - 10 cm^3^ PTV category was the largest. Further inspection of results indicates that for shifts under 1 mm the 5 - 10 cm^3^ size range had the largest increases, but for shifts over 1 mm the 0 - 5 cm^3^ range had the largest, as expected from other results. Additionally, the only decreases in spillage, on average 0.1 ± 0.5%, occurred for the smallest PTVs under 0.25 mm shift.

### Gradient index

**Figure 10:**
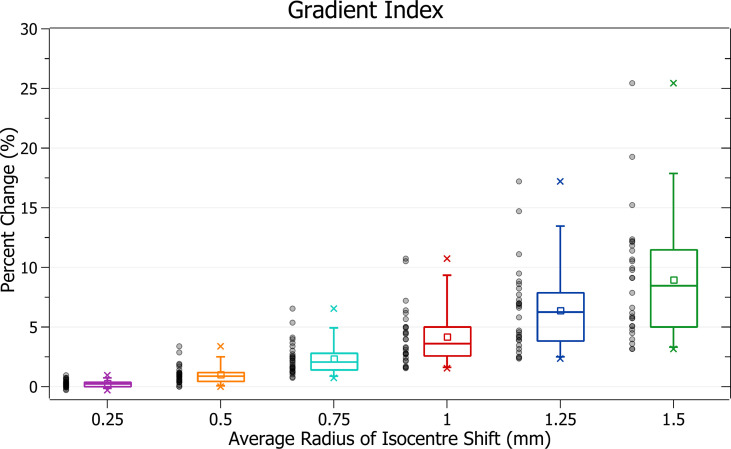
Percentage change of gradient index for different radii of isocentre shift.

**Figure 11:**
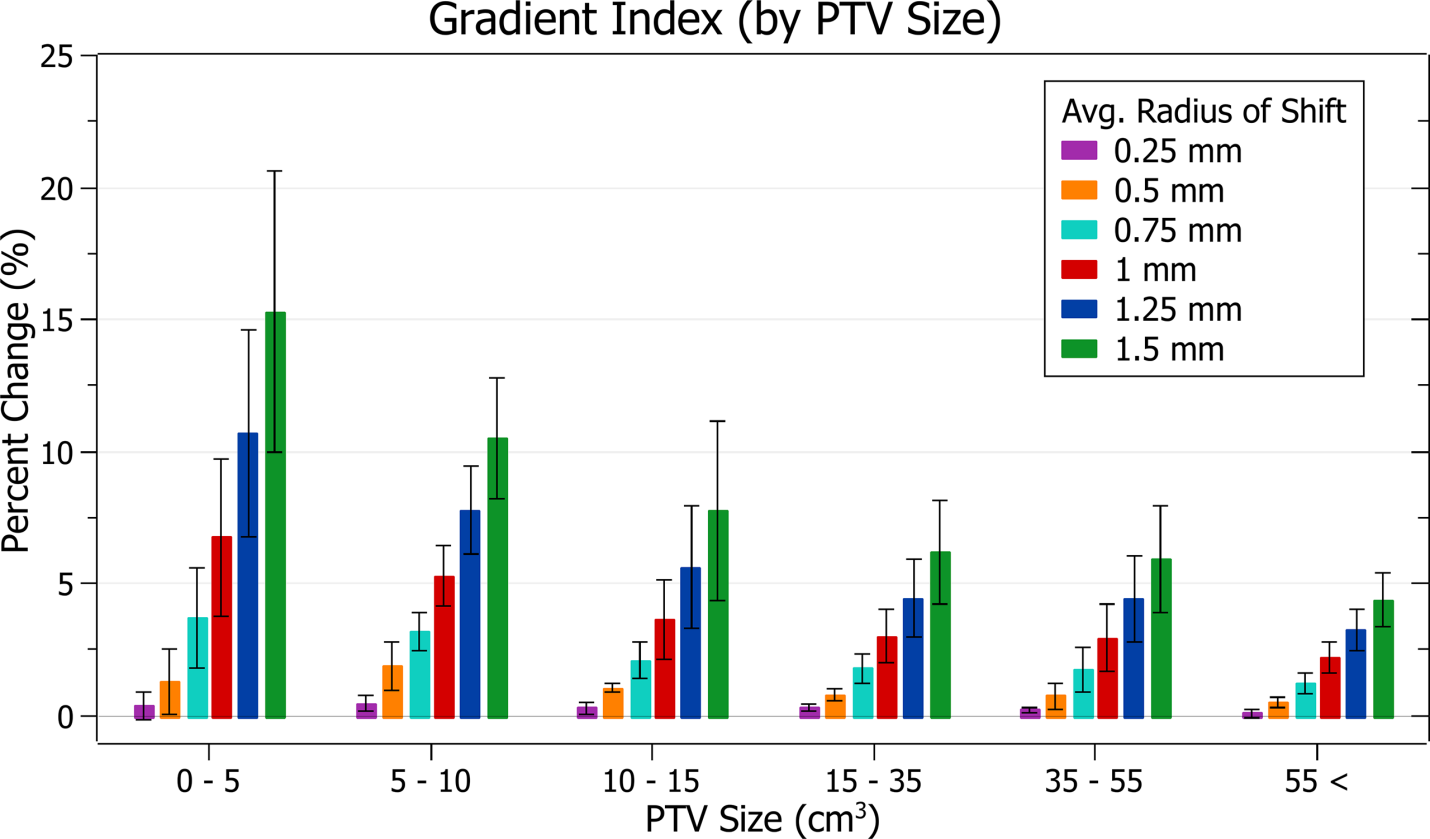
Average percentage change of gradient index for different radii of isocentre shift, categorised by PTV size.

The results of gradient index against radius of isocentre shift are plotted in **Figure 10**. Categorised averages of these shifts are plotted in **Figure 11** for PTV size.

In **Figure 10**, again, plan quality deterioration (i.e. an increasing gradient index) occurs as the average radius of isocentre shift increases. A 0.5 mm shift induces a 1.03 ± 0.77% (P = 1E-7) change, 1 mm: 4.21 ± 2.39% (P = 6E-10) and 1.5 mm: 8.96 ± 5.10% (P = 7E-10). The brain treatments corresponded to the most significant changes.

In **Figure 11**, again small PTV size corresponds with increased isocentre shift sensitivity. At an average shift of 1 mm the percent increase for PTVs between 0 - 5, 5 - 10 and 10 – 15 cm^3^ were 6.8 ± 3.0%, 5.3 ± 1.1% and 3.7 ± 1.5%, respectively.

### Conformity index

**Figure 12:**
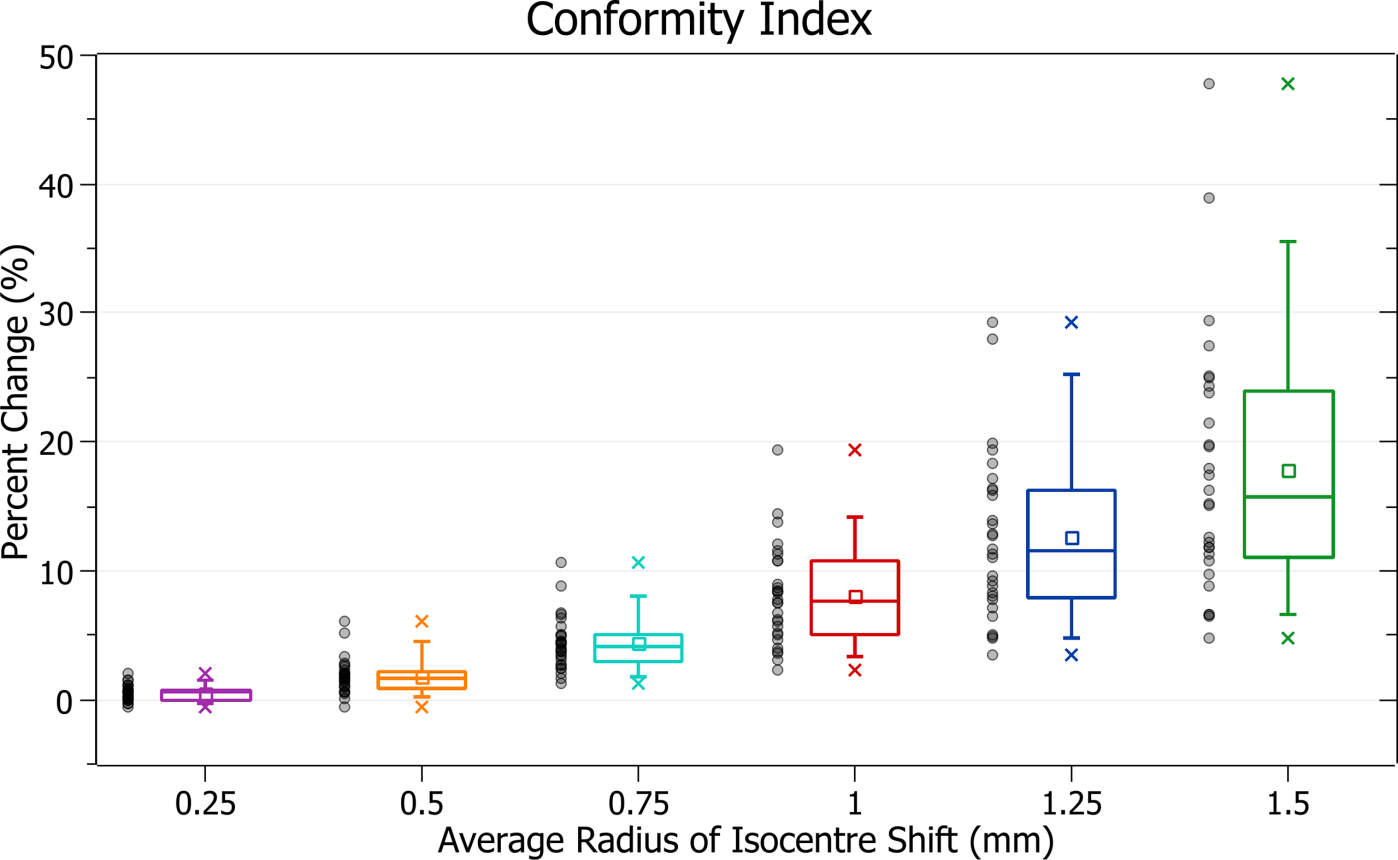
Percentage change of conformity index for different radii of isocentre shift.

**Figure 13:**
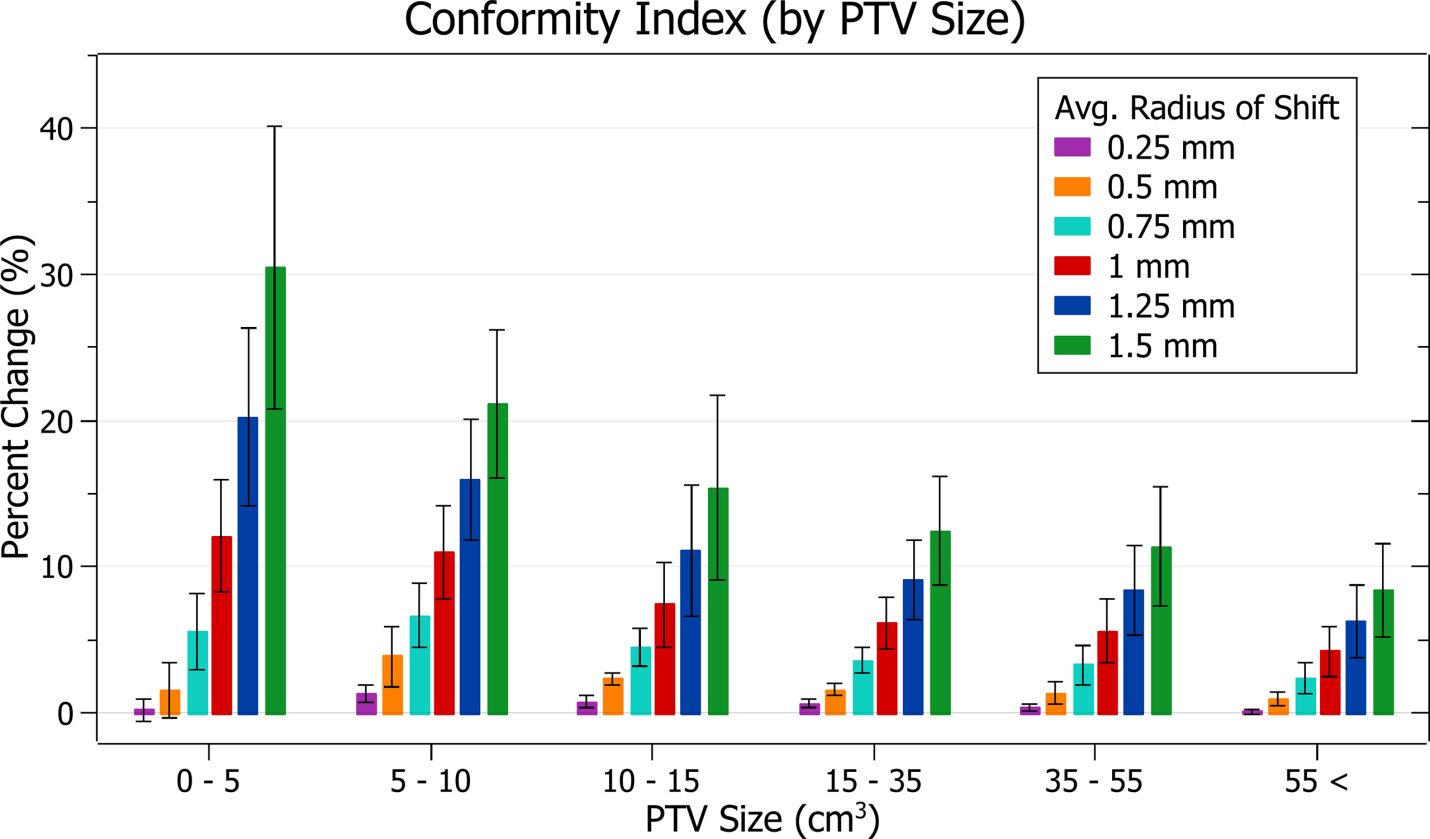
Average percentage change of conformity index for different radii of isocentre shift, categorised by PTV size.

The results of conformity index against radius of isocentre shift are plotted in **Figure 12**. Categorised averages of these shifts are plotted in **Figure 13** for PTV size.

**Figure 12** exhibits the deterioration in plan quality with increased shifts *via* increases in the conformity index. A 0.5 mm shift induces a 1.83 ± 1.38% (P = 2E-7) change, 1 mm: 8.06 ± 3.91% (P = 2E-11) and 1.5 mm: 17.79 ± 10.01% (P = 5E-10). As expected, the brain treatments corresponded with the most significant changes. Notably, the magnitude of all percentage changes was significantly larger than for all other metrics evaluated.

In **Figure 13**, small PTV sizes correlated to increased isocentre shift sensitivity. At an average shift of 1 mm, the percent increase for PTVs between 0 - 5, 5 - 10 and 10 - 15 cm^3^ were 12.1 ± 3.8%, 11.0 ± 3.2% and 7.4 ± 2.9%.

## Discussion

### Validation of beam splitting

As previously noted, perfect congruency was not required between the original and VMAT arc split plans. However, minimal differences were observed across all metrics validating this approach. Primarily, this study was concerned with relative trends against gantry sag derived isocentre shifts, which would not be affected by any variance created during splitting.

Slight deviations were expected due to TPS limitations as rounding was required at two stages of the splitting process. The TPS would not accept angles between 359.9° and 360°, so any interpolated angles falling in this range required adjusting. MLC and gantry angle interpolation also produced higher precision values than those processable by the TPS.

The brain treatment plans exhibited the most significant deviation when split. This coincided with them having the smallest PTVs and, in general, being the most complex plans. A high dose was delivered in a single fraction using 360° arcs in all three of these plans. There was minimal room for error, unlike some of the other plans, which had a combination of larger PTVs, multiple fractions or smaller beam arcs.

### PTV coverage

The PTV coverage exhibited clear trends with radius of isocentre shift and PTV size. PTV coverage decreased with increasing radii of isocentre shift, and small PTV sizes correlated with increased sensitivity to these shifts. The magnitude of these changes and trends agreed with those observed by Wack et al. for static stereotactic treatments (13).

During treatment planning, the beam optimisation process produces complex fluence maps designed to deliver most of the dose to the PTV. Isocentre shifts impact how the fluence from different beams and CPs overlaps, causing the overall fluence map to vary in non-trivial ways. These variations result from beam geometry changes with each beam passing through slightly different tissues. At more significant shifts, the potential magnitude of these variations also increases. In all cases, applying an isocentre shift caused a reduction in coverage except for a small group of plans under 0.25 mm shift. The most significant increase in coverage for any single plan was only 0.3% which may be explained by the stochastic nature of the effect or inaccuracy in dose calculation. It is expected that PTV coverage decreases with increasing isocentre shift.

All average changes were statistically significant, even those caused by 0.25 mm of shift. Clinically, there is no single agreed target for acceptable PTV coverage reduction, and any amount has a probability of decreasing treatment success. A general target used by organisations, such as the AAPM, in developing QA standards, is the ICRU recommendation that the dose delivered be within 5% of that prescribed (11, 18). It is also essential to consider that isocentre variation caused by mechanical uncertainty is only one of many potential sources of error during treatment. Based on the assumption that error in the mechanical isocentre alone should not cause decreases in PTV coverage of more than 4%, the general tolerance level of 1 mm may not be acceptable for all cases considered in this study.

On these assumptions and results, an absolute mechanical isocentre radius tolerance level of 0.5 mm with an action level at 0.75 mm for PTV sizes below 10 cm^3^ is recommended. At PTV sizes above 10 cm^3^, while considering PTV size’s effect on plan performance is recommended, a 1 mm tolerance may be acceptable.

Results support the 0.5 mm recommendation by Wack et al. for static stereotactic treatments. However, this recommendation varies from other thresholds, such as those from TG-142 and TG-198.

### PTV near-minimum

The trends observed in PTV near-min dose relative to prescription dose follow a similar trend to those for PTV coverage.

Although the cause of these trends is the same as for PTV coverage, the reduced sensitivity of near-min dose relative to prescription dose with PTV size may be attributed to slight differences in the metric. It does not account for spatial variation or coverage of a single dose (that prescribed) but rather is a ratio of dose covering the majority of PTV to the prescribed dose.

Clinically, as for a reduction in PTV coverage, the dose near minimum indicates how effectively the prescribed dose is delivered to the PTV. As such, a 4% decrease is again considered the tolerance threshold.

The changes observed in PTV near-min relative to prescription dose support the recommended tolerance levels described in the PTV coverage discussion.

### Prescription dose spillage

The amount of prescription dose spillage increased with increasing radius of average isocentre shift and PTV size was, in general, a good indicator of plan sensitivity to isocentre variance. For shifts under 1 mm, plans with PTV sizes between 0 - 5 cm^3^ exhibited lower spillage than PTV sizes 5 - 10 cm^3^.

The lower prescription spillages for very small PTV sizes and shifts may result from a general reduction in dose. The magnitude and volume of shift-induced dose distribution changes become closer to the PTV size at smaller scales. As effects are not averaged over a large region, it is possible dose changes become more significant, and they induce a more stochastic blurring of dose than that observed for larger PTVs. This could result in a proportionally higher reduction of the volume receiving the prescription dose, especially at the edge of its original isodose line which would generally occur outside the PTV. Therefore, the dose spillage may be the product of a competing reduction in delivered dose and an increased spillage level as it moves outside the PTV. In small PTVs, the overall reduction may become the prevailing effect which could explain why some plans decreased spillage with an isocentre shift. Alternatively, these decreases could result from a dose calculation error at the magnitude observed.

Clinically, in contrast to PTV coverage and near-min metrics, which focus on the PTV, dose spillage provides essential information on the dose delivered to surrounding healthy tissue. Prescription spillage for stereotactic treatment is of significant concern due to the highly damaging doses. The UK SABR Consortium recommends a target of 1.20 and a tolerance of 1.25 for PTV < 20 cm^3^ (15). A hypothetical plan with dose spillage at this target must only change by 4.2% to fall outside the tolerance. A 4% change is thus considered the threshold of acceptability.

Under this assumption, the prescription dose spillage trend further supports the recommended tolerance levels described in the PTV coverage discussion.

### Gradient index

Gradient index trends follow those of the other metrics with a deterioration in plan quality, sensitive to PTV size, and correlated to increasing average radii.

The gradient index indicates unintended doses delivered to healthy tissue like prescription dose spillage. The UK SABR Consortium recommends a target of 7 and a tolerance of 9 for PTV < 20 cm^3^ (15). These limits correspond to a hypothetical plan with a gradient index at target requiring a change of 29% to fall outside tolerance. None of the percentage changes observed for isocentre shifts up to 1.5 mm would cause a change of this magnitude. However, this gradient index tolerance level only relates to what is achievable in treatment planning. It is relatively generous, as the half-prescription dose would not cause as significant damage as the prescription dose. It is also important to note that specific organ at risk (OAR) constraints, rather than a general dose delivered to healthy tissue, are required to evaluate clinical outcomes.

For a theoretical prescribed dose of 20 Gy, the gradient index relates to the volume receiving at least 10 Gy. Although there is significant variation in literature, a dose of 10 Gy is close to or above most single fraction constraints for various OARs, such as the spinal cord and lung (19). While the gradient index does not provide information on the exact location of the half-prescription isodose line, it relates to a clinically significant dose.

At 1 mm of isocentre shift for PTVs between 0 and 5 cm^3^, a 6.8 ± 3.0% increase in the gradient index was observed. The change drops to 3.7 ± 1.9% for 0.75 mm of shift. Any increase in dose to healthy tissue increases plan complication risk; however, the clinical impact of these values is dependent on OAR locations. Despite this, given the significant reduction of change in lowering the tolerance to 0.75 mm, the recommended tolerance levels described in the PTV coverage discussion are likely to improve patient outcomes.

### Conformity index

The conformity index was the most sensitive metric of those examined, and significant changes were observed with increasing average radii of shift. Trends with PTV size were also in agreement with other metrics.

The conformity index is a metric that considers both PTV coverage and prescription dose spillage making it particularly sensitive. It displayed the most considerable changes in a trend consistent with other metrics evaluated.

As the conformity index can be considered a combination measure of PTV coverage and spillage, for which a 4% change was considered the tolerance level, an 8% change was considered significant.

Based on this level, the observed conformity index trends agree with other metrics in supporting the recommended tolerance levels described in the PTV coverage discussion.

### Limitations

75% of the plans used in this study were for pelvic bone treatment sites, with only seven at other locations. While overall trends should still generalise to other treatment sites, bone has a high density which would produce different dose distributions to those for volumes with lower density tissue. However, most analyses were conducted relative to the distribution of a given plan, and conclusions are not reliant on any particular distribution.

The DVH derived metrics reported also did not directly consider the dose delivered to and coverage of OARs, which may be close to the PTV. It is essential to consider these during treatment planning as too much dose to an OAR can lead to potentially severe complications. While these metrics provide a general overview of plan quality, clinicians should ensure they also examine the dose distribution itself. Dose conformity and spillage metrics indicate the quantity of dose delivered to surrounding healthy tissue, but do not specifically evaluate it for individual OARs. Although the lack of immediate OAR consideration is unlikely to have a significant impact on general conclusions, for plans with an OAR close to the PTV, such as in the brain, even tighter tolerance levels may be required.

A further study considering a more comprehensive range of plan quality parameters and treatment sites may help validate the general applicability of these results and conclusions.

### Clinical impacts and potential implementation

In evaluating the dosimetric impact from isocentre shifts through five clinically relevant metrics, a recommended absolute mechanical isocentre radius tolerance level of 0.5 mm with action level at 0.75 mm for PTV sizes below 10 cm^3^ is suggested. This tolerance is achievable in modern linacs. In treatments of PTV sizes above 10 cm^3^ a 1 mm tolerance may be acceptable; however, considering the effect PTV size may have on plan performance is still recommended. Constraining the isocentre tolerance is one avenue to address these effects. However, this may not be possible in some centres, especially if older machines are used.

The clinical impact of these findings is twofold. Firstly, PTV coverage and PTV near-minimum dose are surrogates for absorbed dose to the tumour. Reductions would be expected to translate to reduced tumour control probability and the potential for poorer disease outcomes. Whilst this could be compensated by increasing the PTV margin, this is very difficult in practice when a tumour is close to critical structures (such as the brain or spinal cord). OAR constraints are typically not breached due to potential catastrophic complications; ultimately, the target PTV doses would be preferentially compromised. Although the focus of the present work was on PTV dose parameters, what matters clinically is the GTV coverage. However, a direct reading of GTV coverage from the treatment planning system does not account for effects such as intra- and inter-fraction motion and MLC interplay, and therefore has little clinical relevance. In particular, the results in **Table S1**, which suggest that GTV coverage remains largely unaffected, apply only if gantry sag was the sole perturbation (for the GTV-PTV margins listed in **Table 1**). As the scope of this research is limited to the effect of gantry sag, this study focuses on the PTV to allow the PTV margin to account for such motion effects.

The second immediately clinically relevant impact of this source of mechanical error is the inferred greater dose to surrounding structures, potentially as high as the prescription dose or even higher. Small variations in dose received by critical structures, such as the brain and spinal cord, at common stereotactic doses per fraction can arguably be the difference between safe treatment and significant toxicity. A bigger dose per fraction to a portion of an OAR also means a higher total dose received (if multiple fractions) with a supra-additive radiobiological impact (double-trouble phenomenon).

Radionecrosis of brain tissue, myelopathy from spinal cord injury and perforation of gastrointestinal viscera are all potentially lethal sequelae of stereotactic radiotherapy resulting from excessive dose to nearby OARs. Perhaps the most common site of significant stereotactic radiotherapy injury is within the brain where the risk of symptomatic radionecrosis is as high as 10% in some series (asymptomatic rates up to 30%) (20). With greater recognition and correction of uncertainties, such as the described isodose deviations during a treatment arc, it may be possible that such toxicity can be reduced.

An alternate option, for machines unable to meet the proposed isocentre tolerances, is to directly correct the specific mechanical errors associated with a linac by adjusting for them during treatment planning. A previous study by Du et al. indicates that a correction strategy can reduce gantry sag to less than 0.2 mm (5). The ability to do this depends on the planning system since it may not be possible in some. The full angular dependent isocentre shifts would need to be quantified. However, a potential benefit of such an approach is the accounting of machine-specific characteristics. Such an approach has been reported as clinically possible for static stereotactic treatment (21).

In a clinical situation where none of the above solutions can be implemented, and mechanical isocentre variance is larger than recommended tolerance levels, the machine may not be suitable for stereotactic treatment. In these situations, a patient may be transferred to a different machine or referred to a different centre capable of more accurately delivering stereotactic treatments. In some cases (*e.g.* limited brain metastases), riskier treatments such as surgery could also be considered. As an alternative, fractionated treatments would allow a level of compensation for this error between fractions. The use of lower doses while removing the heightened risks associated with high doses would also remove their associated radiobiological benefits. Non-radiotherapy methods, such as chemotherapy, may also be more suitable than the risk of delivering stereotactic treatment with a high level of uncertainty.

## Conclusion

This study investigated the dosimetric effect of varying magnitudes of mechanical inaccuracy during linac gantry rotation for 28 stereotactic VMAT plans. A VMAT beam splitting algorithm was created to enable the precise application of gantry sag derived isocentre variation at each CP angle within a dynamic treatment arc. Dose distributions were simulated using a clinically validated TPS, and five plan evaluation metrics were extracted.

Significant plan deterioration was observed for small PTVs at the 1 mm mechanical isocentre tolerance level commonly recommended in the literature. Based on the results presented, an alternative tolerance level of 0.5 mm with an action level of 0.75 mm is recommended for PTV sizes below 10 cm^3^. If this is not achievable, alternatives should be considered, such as explicitly including this error in the PTV margin, directly accounting for machine-specific error during planning or using a different cancer treatment approach.

Due to potential variation with treatment sites, it is desirable to see a similar analysis conducted at a broader range of treatment sites. Future work could also examine the impacts of other sources of machine inaccuracy, such as MLC resolution, couch sag and MLC carriage sag. Dose delivery accuracy through effective QA is critical to patient outcomes, and this study provides essential quantitative data to inform QA practice development.

## Data availability statement

The original contributions presented in the study are included in the article/**Supplementary Material**. Further inquiries can be directed to the corresponding author.

## Ethics statement

The ethics approval for this project was granted by the Sir Charles Gairdner and Osborne Park Hospital Group as a Quality Improvement Activity (44128).

## Author contributions

BM-H, PR, and TM contributed to the conception and design of the study. BM-H performed plan evaluation and analysis. All authors discussed the results. RW provided clinical insights. BM-H wrote the first draft of the manuscript. All authors contributed to the article and approved the submitted version.

## Acknowledgments

We thank the Department of Radiation Oncology, Sir Charles Gairdner Hospital, for providing plan data and access to Varian Eclipse.

## Conflict of interest

The authors declare that the research was conducted in the absence of any commercial or financial relationships that could be construed as a potential conflict of interest.

## Publisher’s note

All claims expressed in this article are solely those of the authors and do not necessarily represent those of their affiliated organizations, or those of the publisher, the editors and the reviewers. Any product that may be evaluated in this article, or claim that may be made by its manufacturer, is not guaranteed or endorsed by the publisher.
