# Supplementary material for: Dosimetric impact of mechanical movements of the Linac gantry during treatments with small fields
Source: Front Oncol. 2022 Nov 3;12:973431. doi: 10.3389/fonc.2022.973431 (PMC9669569; doi:10.3389/fonc.2022.973431)
Supplement: Supplementary file 1 [file DataSheet_1.pdf]

Supplementary Material

Dosimetric Impact of Mechanical Movements of the Linac Gantry During Treatments with Small Fields

Broderick McCallum-Hee[1*], Thomas Milan[2], Rohen White[2], Pejman Rowshanfarzad[1]

[1]School of Physics, Mathematics and Computing, The University of Western Australia, Crawley, WA 6009, Australia

[2]Department of Radiation Oncology, Sir Charles Gairdner Hospital, Nedlands, WA 6009, Australia

*** Correspondence:**
Broderick McCallum-Hee
Broderick.hee@outlook.com

1 Supplementary Figures

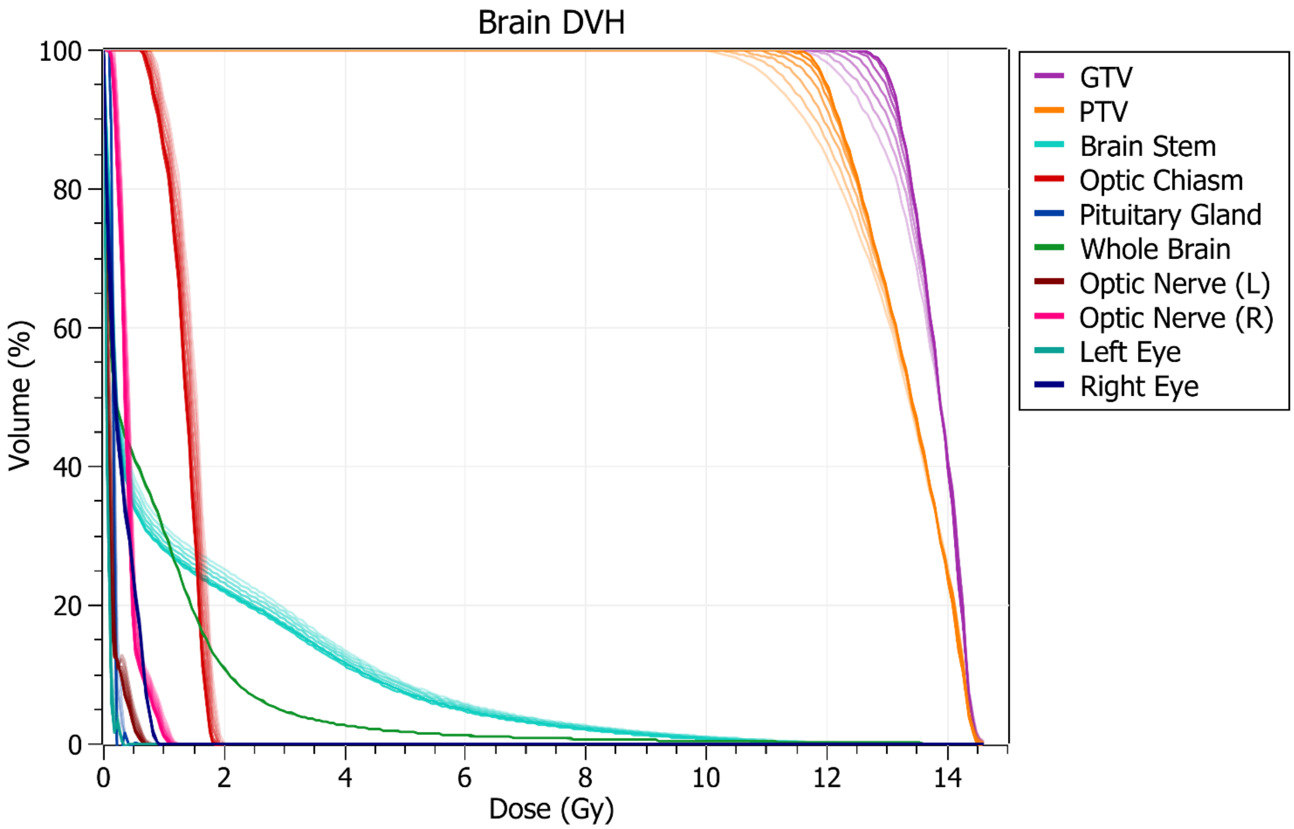

Figure S1. DVH for a brain treatment plan at different radii of isocentre shift (0, 0.25, 0.5, 0.75, 1, 1.25 and 1.5 mm). Opacity of the curves is reduced as the isocentre shift increases.

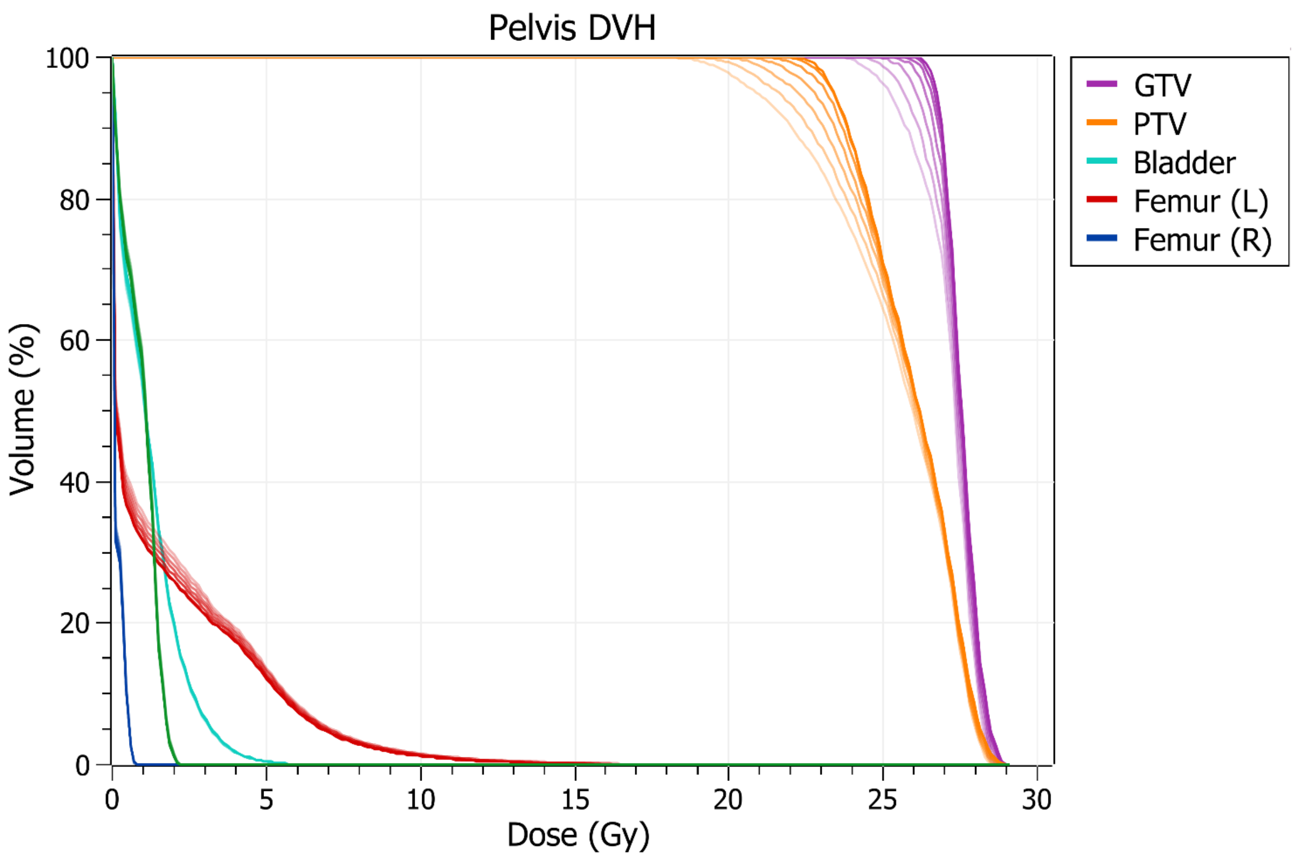

Figure S2. DVH for a pelvis treatment plan at different radii of isocentre shift (0, 0.25, 0.5, 0.75, 1, 1.25 and 1.5 mm). Opacity of the curves is reduced as the isocentre shift increases.

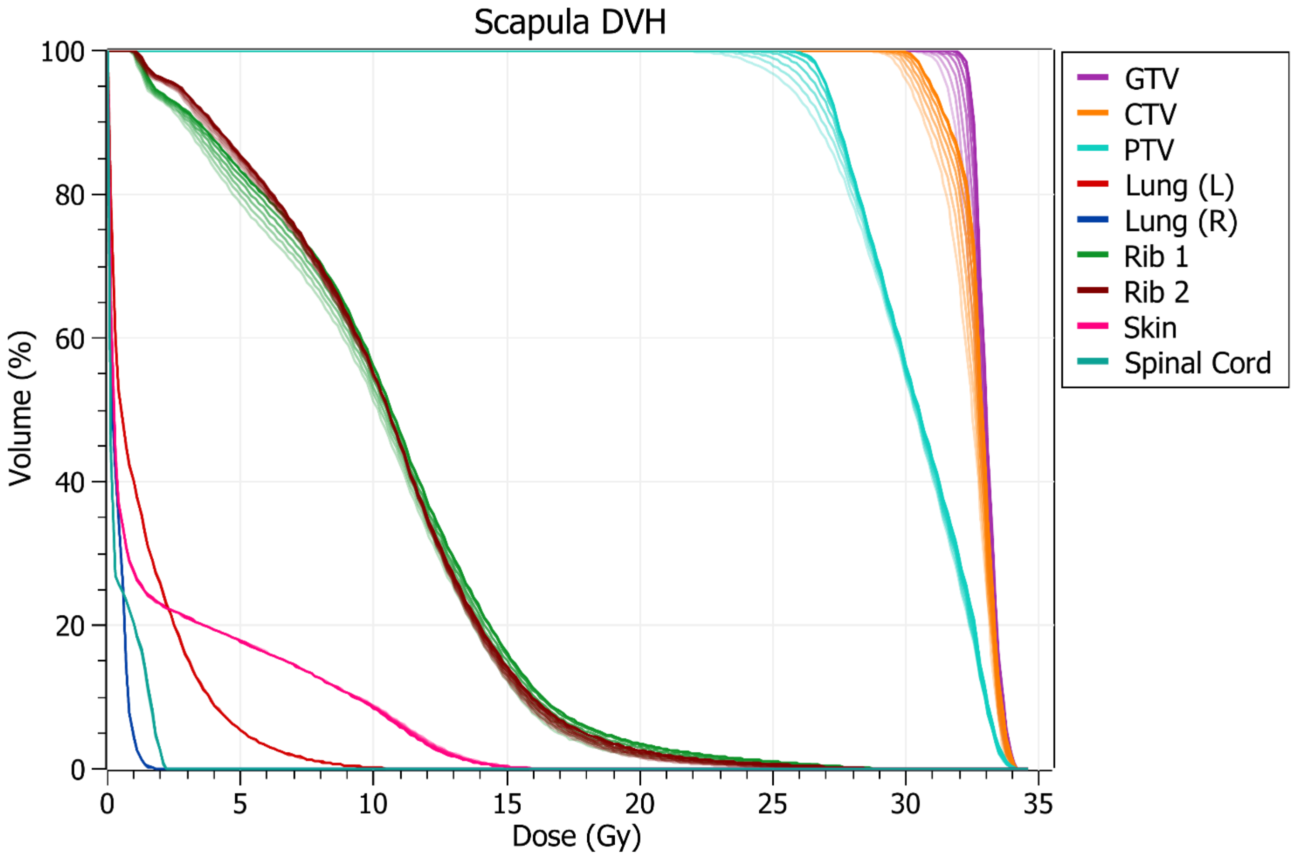

Figure S3. DVH for a scapula treatment plan at different radii of isocentre shift (0, 0.25, 0.5, 0.75, 1, 1.25 and 1.5 mm). Opacity of the curves is reduced as the isocentre shift increases.

2 Supplementary Tables

Table S1: Summary of all plans used in this study grouped by PTV size with GTV Coverage after application of an isocentre shift with average radius 1.5 mm.

**PTV Category**	**GTV** (cm^3)	**CTV** (cm^3)	**PTV** (cm^3)	**Treatment Site**	**GTV Coverage After Application of 1.5 mm Avg. Isocentre Shift** (%)
0 - 5	-	-	1.41	Brain	-
	0.41	-	2.4	Brain	100
	1.62	-	2.78	Brain	**98.14**
	0.21	-	3.04	Pelvis - Iliac (Right)	100
	0.2	-	3.16	Pelvis - Sacrum	100
	0.58	-	3.22	Femur (Left)	100
	1.51	-	4.41	Pelvis - Iliac (Left)	**99.63**
5 - 10	0.54	-	5.64	Pelvis - Ilium (Right)	100
	1.38	3.25	6.72	Pelvis - Pubis (Right)	100
	1.21	4.34	8.38	Pelvis - Ilium (Left)	100
10 -15	0.61	5.03	11.81	Pelvis - Ischium (Left)	100
	2.37	6.37	11.93	Pelvis - Ilium (Right)	100
	3.63	-	12.89	Pelvis - Iliac (Left)	100
	1.47	4.95	14.11	Pelvis - Ischium (Left)	100
	1.68	4.79	14.78	Scapula (Right)	100
15 - 35	10.73	-	20.85	Pelvis - Iliac (Left)	**99.99**
	5.63	9.98	20.97	Pelvis - Iliac (Right)	100
	13.07	-	24.03	Pelvis - Iliac (Right)	**99.67**
	1.7	17.67	33.56	Pelvis - Ischium (Left)	100
	1.89	18.38	34.84	Pelvis - Pubic Ramus (Left)	100
35 - 55	14.2	-	40.62	Pelvis - Ischium (Left)	100
	21.17	-	40.75	Pelvis - Sacrum	100
	3.85	25.75	45.8	Pelvis - Pubis (Left)	100
	28.12	-	47.29	Pelvis - Sacrum (Nodes)	**99.98**
	9.3	12.14	47.91	Scapula (Left)	100
55 +	19.39	34.84	58.95	Pelvis - Sacrum	100
	12.62	51.51	85.24	Pelvis - Acetabulum (Left)	100
	5.14	56.79	89.09	Neck of Femur (Left)	100
